# Cognitive change in prevalent and incident hearing loss: The Maastricht Aging Study

**DOI:** 10.1002/alz.13606

**Published:** 2024-01-18

**Authors:** Lion M. Soons, Kay Deckers, Huibert Tange, Martin P. J. van Boxtel, Sebastian Köhler

**Affiliations:** ^1^ Alzheimer Centrum Limburg Department of Psychiatry and Neuropsychology School for Mental Health and Neuroscience (MHeNs) Maastricht University Maastricht The Netherlands; ^2^ Care and Public Health Research Institute (CAPHRI) Department of Family Medicine Faculty of Health Medicine and Life Sciences Maastricht University Maastricht The Netherlands

**Keywords:** audiometry, cognitive decline, dementia, executive function, hearing acuity, hearing loss, information processing speed, memory, modifiable risk factors, prevention, risk reduction

## Abstract

**INTRODUCTION:**

Hearing loss (HL) has been associated with cognitive decline and dementia. We examined the temporal association between prevalent and incident HL and cognitive change.

**METHODS:**

A total of 1823 participants (24‐82 years) from the Maastricht Aging Study (MAAS) were assessed at baseline, 6 and 12 years, including pure‐tone audiometry. Linear‐mixed models were used to test the association between HL and cognition, adjusted for demographics and other dementia risk factors.

**RESULTS:**

Participants with prevalent and incident HL showed a faster decline in verbal memory, information processing speed, and executive function than participants without HL. Decline was steady from baseline to 6 and 12 years for prevalent HL, but time‐delayed from 6 to 12 years for incident HL. Having a hearing aid did not change associations.

**DISCUSSION:**

Findings support the notion that HL is a risk factor for cognitive decline independent of other dementia risk factors. Onset of HL preceded onset of cognitive decline.

**Highlights:**

- We examined cognitive change in prevalent and incident hearing loss.
- Prevalent and incident hearing loss were associated with faster cognitive decline.
- For prevalent hearing loss, decline was steady from baseline to 6 and 12 years.
- Onset of hearing loss preceded the onset of cognitive decline.
- Having a hearing aid did not change the observed associations.

## 1 BACKGROUND

The prevalence of dementia is increasing globally from 57 million in 2019 to more than 153 million by 2050, making it a public health priority.^1^ To date, the most promising strategies against the rising dementia prevalence and associated burden imposed on people living with dementia, as well as their caregivers, families, and society, consist of developing disease‐modifying pharmacological treatments and lifestyle‐based dementia risk reduction programs.^2^ According to the 2020 report of the Lancet Commission on Dementia Prevention, Intervention and Care, about 40% of dementia cases are potentially attributable to 12 modifiable risk factors. Midlife hearing loss (HL) showed the highest population attributable fraction (9%), which reflects the percentage reduction of new dementia cases if midlife HL would be completely eliminated.^3^

Various systematic reviews have concluded that people with HL have a higher risk of cognitive impairment and dementia.^4^, ^5^ In addition, HL is also highly prevalent in later life and, therefore, reducing HL prevalence has the potential to be of significant public health impact, if the association with cognitive decline is causal. The World Health Organization (WHO) estimates that 65% of individuals aged more than 60 years have some degree of HL (≥20 decibels (dB) at the better ear). Nearly 25% of them experience moderate to profound HL, also referred to as hearing disability (≥35 dB). The risk of disabled HL increases with age, with approximately 13% of individuals at the age of 60 years having a hearing disability, and over 58% at 90 years.^6^ However, despite the age‐related prevalence of HL, the Lancet Commission has identified HL as an important risk factor already in midlife.^3^ Therefore, studies investigating the association between HL and cognitive decline and dementia in cohorts with a broad age range covering both midlife as later life are needed.

Understanding the relation between HL and cognitive decline and dementia is important because it provides opportunities for timely risk management using available rehabilitative hearing interventions, such as hearing aids. Indeed, some observational studies have found that the association between HL and cognitive impairment or dementia is only present in those not using hearing aids.^7^, ^8^ Others found that episodic memory performance seems to deteriorate less in individuals after they start using hearing aids.^9^ However recently, results from the ACHIEVE trial, which is the first large, randomized controlled trial (RCT) examining the effect of hearing aids on global cognitive decline over 3 years, showed no effect in the main analyses. Only in a subsample of individuals with cardiovascular risk, a significant difference between groups was found.^10^ Earlier RCTs on the effect of hearing rehabilitation on cognitive function are sparse, inconclusive, and limited in follow‐up or sample size,^11^ limiting causal inference. In addition, no study has focused on the onset and course of cognitive functioning in individuals with incident HL. Such studies would provide important insight into the temporal relationship between onset of risk exposure and onset of cognitive decline, inform about reverse causation (i.e., onset of cognitive decline preceding onset of HL), and inform about the existence of a (time) window for risk management.

To validate and build upon previous research, we examined the association between both prevalent and incident HL and cognitive decline in a large prospective cohort study covering the whole adult age range with comprehensive data on cognitive functioning and hearing ability. The primary aim was to compare the cognitive trajectories of individuals with prevalent and incident HL to those without HL in the general adult population. We also assessed whether the association was independent of other modifiable dementia risk factors and studied potential effect modification by age and hearing aid. We hypothesize that prevalent and incident HL are associated with accelerated cognitive decline, independently of other modifiable dementia risk factors and more pronounced at higher age. Furthermore, we expect that among those with HL, cognitive decline was faster for individuals who did not have hearing aids.

**RESEARCH IN CONTEXT**

1. **Systematic review**: We reviewed the literature on hearing loss and cognitive decline using traditional medical databases (e.g., PubMed). Although epidemiological studies have found support for an association between hearing loss and cognitive decline, no study focused on the onset and course of cognitive functioning in incident hearing loss.
2. **Interpretation**: Our findings support the notion that prevalent hearing loss is associated with faster cognitive decline, and adds to earlier findings that this is also true for incident hearing loss. In incident hearing loss, onset of hearing loss preceded the onset of cognitive decline, which possibly opens a window for risk management. Having a hearing aid did not change the associations.
3. **Future directions**: Our findings encourage randomized controlled trials to examine the effect of hearing aid use on cognitive functioning and inform about causality of associations. Increasing awareness and promoting accessibility and use of hearing aids may have important public health implications.

## 2 METHODS

### 2.1 Study sample and design

Data were used from the Maastricht Aging Study (MAAS), a prospective cohort study on determinants of cognitive aging in community‐dwelling adults.^12^ Participants for MAAS were recruited from the Registration Network Family Practices (RNFM, formerly known as RNH), a patient register of collaborating general practitioners in the Province of Limburg, the Netherlands. Patients in the RNFM are representative of the population in Limburg with respect to demographic characteristics.^13^

A total of 10,396 patients were invited from the RNFM to participate in MAAS. Individuals were excluded if evidence of major neurological conditions or psychiatric disorders known to interfere with normal cognitive function was present in the patient records, such as dementia, a history of coma, cerebrovascular pathology, or epilepsy. All potentially eligible participants were then screened in a semi‐structured interview by telephone to make sure that no exclusion criteria were overlooked. Subsequently, a sub‐sample of 1823 individuals was drawn using an optimal stratified sampling design with equally balanced strata for age (12 discrete age groups from 24 to 81 years), sex, and level of occupational achievement (low/high). At baseline (1993‐1996), participants underwent a comprehensive assessment of medical status, lifestyle, and anthropomorphic and neurocognitive measures. These measures were repeated 6 and 12 years after baseline.^12^ The local ethics committee of Maastricht University Medical Centre approved the study (MEC05‐107). All participants gave informed consent.

### 2.2 Hearing assessment

Hearing was assessed at baseline, 6‐ and 12‐year follow‐up. Pure‐tone audiometry thresholds were measured at each ear separately at frequencies of 0.5, 1, 2, and 4 kilohertz (kHz), using a screening audiometer (Interacoustics AS7, Denmark). Detection thresholds were determined in steps of 10 dB, with 0 dB as the minimum and 90 dB as the maximum stimulus intensity.^12^ According to recommendations for the assessment of hearing handicap, overall hearing acuity was expressed as the average of hearing thresholds at 1, 2 and 4 kHz for the better, unaided ear,^14^ with higher values reflecting worse hearing acuity. Subsequently, in order to identify individuals at risk, prevalent HL (at baseline) was categorized into no HL (loss < 20 dB), mild HL (loss ≥ 20 and < 35 dB) and moderate to profound HL (loss ≥35 dB), based on the recent WHO guidelines.^6^ Incident HL was defined binarily (yes/no) as having at least mild HL at 6‐ or 12‐year follow‐up in those without baseline HL. Having hearing aids were assessed at 6‐year follow‐up by asking participants whether they had hearing aids and year of prescription. Based on the date of the baseline visit, we determined whether individuals already possessed hearing aids at baseline.

### 2.3 Cognitive assessments

Psychologists and trained test assistants administered a neuropsychological test battery at baseline, 6‐ and 12‐year follow‐up. The delayed recall of the Visual Verbal Learning Test was used to assess verbal memory.^15^ A set of 15 non‐related monosyllabic words were presented in each of five subsequent trials on a computer screen, followed by immediate (after each trial) and delayed (20 min after last trial) recall phases. The Concept Shifting Test was used to assess executive function.^16^ In three consecutive trials, the participant had to cross out digits in ascending order (part A), letters in alphabetic order (part B), and finally digits and letters in alternating order (part C) as fast as possible. Subsequently, a shifting score was calculated by subtracting the average time needed to complete part A and B from the time needed to complete part C. To assess information processing speed, the Letter Digit Substitution Test was used.^17^ Using a reference key, the participant had 90 seconds to match specific digits to given letters as quickly as possible.

### 2.4 Covariates

Age, sex, and educational level (categorized into low, middle, and high) were included as demographic covariates. Modifiable lifestyle factors at baseline were summarized based on the “LIfestyle for BRAin health” (LIBRA) index.^18^ The LIBRA index is a well‐validated compound score that has been associated with cognitive functioning/decline, cognitive impairment, brain damage on neuroimaging and incident dementia in several population‐based cohort studies,^19^, ^20^, ^21^ and has been suggested as a (surrogate) outcome measure in prevention trials.^22^, ^23^ It is based on the weighted contributions (positive for risk factors, negative for protective factors) of 12 risk and protective factors for dementia, that is, lifestyle factors and somatic conditions: high cognitive activity, physical inactivity, adherence to the Mediterranean diet (not available/measured in MAAS), smoking, low‐to‐moderate alcohol consumption, hypertension, hypercholesterolemia, obesity, diabetes, coronary heart disease, chronic kidney disease, and depression. The total LIBRA score ranges from −5.9 to +12.7, with higher scores representing an unhealthier lifestyle and higher dementia risk. In MAAS, information was available for all LIBRA factors at baseline, except for the protective factor Mediterranean diet, yielding a theoretical range of −4.2 to +12.7. Detailed information on the operationalization of the individual risk factors for the LIBRA index in MAAS can be found in Supplementary Table 1.

### 2.5 Statistical analyses

Differences in[Table alz13606-tbl-0001] demographic information and LIBRA scores between participants with and without prevalent/incident HL were analyzed using independent samples t‐tests and χ^2^‐tests. One data transformation was used for the negatively skewed distribution of the Visual Verbal Learning Test (square root transformation). We used Linear‐mixed models to test the association between hearing acuity, prevalent, and incident HL and change in cognition over time (for each cognitive domain separately). As suggested by likelihood ratio testing, models including a random intercept and random slope with an unstructured covariance matrix had the best fit and were subsequently used. An interaction term was added to model the association between HL and cognitive decline (HL x time), with time entered as a dummy variable for the two follow‐ups (1 = baseline to 6 years; 2 = baseline to 12 years). The interaction terms were tested using a χ^2^‐test with 2 degrees of freedom.

The analytical approach was as follows: First, analyses were performed for (continuous) baseline hearing acuity (in dB). Second, they were repeated using prevalent HL as a categorical variable. Third, analyses were run replacing prevalent by incident HL. Fourth, to investigate the influence of hearing aids on the association between prevalent HL and cognitive decline, HL was grouped into three categories: no HL, HL with hearing aids, and HL without hearing aids. In addition, three‐way interactions were tested using a χ^2^‐test with 2 degrees of freedom to study potential effect modification by age group (<65 years and ≥65 years) on the association between prevalent and incident HL and cognition over time, followed by stratified analyses in case of statistical significance. All analyses were adjusted for age, age^2^ (to account for non‐linearity), sex, and level of education, with the fully adjusted model also including the LIBRA scores and LIBRA by time interaction. Final results are shown from the fully adjusted model. To reduce selection bias, two inverse probability weights were used. First, a sampling weight was added to weight back the estimates to the RNFM population—the sample frame of MAAS. The sampling weight was based on a probit regression with age, sex, and level of occupational achievement as predictors, so that participants who were less likely to be sampled were given more weight in the analyses. Second, an attrition weight containing information on the probability for a given participant of having missing data was used. These participants were given more weight in the analyses, minimizing bias due to selective attrition. All analyses were performed in Stata 17 (StataCorp, TX) with an alpha level of .05 in two‐tailed tests.

## 3 RESULTS

### 3.1 Participant characteristics

**FIGURE 1:**
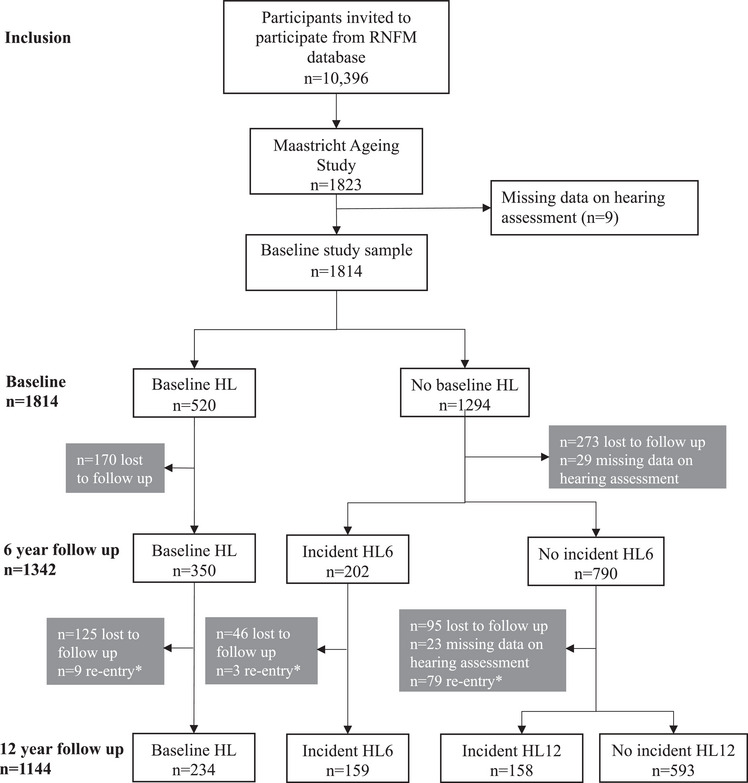
Flowchart of the study population. RNFM, Research Network Family Medicine Maastricht; MAAS, Maastricht Aging Study; HL, hearing loss. *Individuals lost to follow‐up at year 6 could re‐enter the study sample at year 12.

A flowchart of the study population is shown in Figure 1.[Table alz13606-tbl-0002], [Table alz13606-tbl-0003]

Table 1 presents the participant characteristics of the sample by HL status. Of 1823 participants, 9 were excluded due to missing hearing data at baseline. The remaining 1814 participants all had available data on at least one cognitive outcome at baseline. Of those, 322 (17.8%) participants were classified with mild prevalent HL and 198 (10.9%) were classified with moderate to profound prevalent HL. Participants with mild or moderate to profound prevalent HL were on average older, less often female, had a lower education level, a higher (i.e., unhealthier) LIBRA score, lower performance on cognition tasks and had more often hearing aids than those without prevalent HL. During follow‐up, 360 (33.6%) individuals were classified with incident HL. Similar to those with prevalent HL, they were on average older, less often female, had a lower education level, and lower performance on cognition tasks than those without incident HL.

**TABLE 1 alz13606-tbl-0001:** Baseline characteristics by hearing loss status at baseline and follow‐up.

	Prevalent HL (*n* = 1814)			Incident HL (*n* = 1071)			
Characteristic	No, *n* = 1294	Mild, *n* = 322	Moderate‐profound, *n* = 198	*P‐*Value	No, *n* = 711	Yes, *n* = 360	*P‐*Value
Age, mean (SD)	45.6 (14.4)	64.1 (10.6)	70.4 (8.8)	<0.001	39.5 (11.3)	54.6 (11.1)	<0.001
Female, n (%)	696 (53.8)	143 (44.4)	69 (34.9)	<0.001	386 (54.3)	169 (46.9)	0.023
**Education level, n (%)**				<0.001			<0.001
Low	394 (30.5)	161 (50.3)	106 (53.5)		159 (22.4)	158 (43.9)	
Middle	566 (43.7)	114 (35.6)	64 (32.3)		341 (48.0)	139 (38.6)	
High	344 (25.8)	45 (14.1)	28 (14.1)		211 (29.7)	63 (19.5)	
Hearing acuity, mean (SD)	6.8 (5.5)	25.8 (4.2)	45.1 (8.8)	<0.001	4.0 (3.7)	11.3 (4.8)	<0.001
Hearing aid use, n (%)	0 (0)	3 (0.9)	29 (14.6)	0.005	n.a.	n.a.	n.a.
**LIBRA factors, n (%)**							
Hypertension	254 (19.6)	159 (49.4)	116 (58.6)	<0.001	79 (11.1)	109 (30.3)	<0.001
Diabetes	38 (2.9)	22 (6.8)	21 (10.6)	<0.001	10 (1.4)	15 (4.2)	0.005
Hypercholesterolemia	104 (8.0)	49 (15.2)	20 (10.1)	<0.001	37 (5.2)	44 (12.2)	<0.001
Coronary heart disease	91 (7.0)	69 (21.4)	47 (23.7)	<0.001	31 (4.4)	38 (10.6)	<0.001
Chronic kidney disease	56 (4.3)	15 (4.7)	9 (4.6)	0.962	31 (4.4)	9 (2.5)	0.129
Obesity	197 (15.2)	74 (23.0)	57 (28.8)	<0.001	89 (12.5)	64 (17.8)	0.021
Smoking	397 (30.8)	87 (27.5)	40 (20.3)	0.009	224 (31.6)	93 (25.8)	0.051
High cognitive activity	359 (27.0)	141 (44.1)	91 (46.2)	<0.001	139 (19.6)	126 (35.1)	<0.001
Physical inactivity	453 (35.1)	134 (42.1)	91 (46.7)	0.001	254 (35.8)	117 (32.8)	0.331
Low‐to‐moderate alcohol use	753 (59.9)	203 (65.7)	122 (65.2)	0.089	413 (59.3)	203 (58.7)	0.857
High depressive symptoms	306 (23.7)	71 (22.4)	46 (23.8)	0.885	173 (24.3)	86 (24.0)	0.892
LIBRA score, mean (SD)	0.7 (2.2)	1.0 (2.5)	1.2 (2.6)	0.009	0.7 (2.1)	0.7 (2.4)	0.775
**Neuropsychological tests, mean (SD)**							
Verbal Learning Test (raw)	10.3 (2.8)	8.6 (3.1)	7.4 (2.9)	<0.001	10.9 (2.5)	9.4 (2.8)	<0.001
Verbal Learning Test (transformed)	113.2 (55.4)	83.8 (52.1)	63.3 (44.8)	<0.001	125.6 (52.7)	96.3 (52.4)	<0.001
Letter Digit Substitution Test (raw)	51.4 (10.9)	41.5 (10.1)	38.9 (9.6)	<0.001	54.6 (9.9)	46.8 (10.5)	<0.001
Concept Shifting Test (raw)	10.5 (9.8)	16.2 (14.3)	16.4 (12.5)	<0.001	8.6 (7.6)	12.5 (10.1)	<0.001

*Note*: Means, SDs, and percentages are back‐weighted to the sampling frame. Percentages may not sum up to 100% because of rounding errors.

Abbreviations: BEPTA, best ear pure tone audiometry; HL, hearing loss; LIBRA, LIfestyle for BRAin health index (theoretical range −4.2 to +12.7); SD, standard deviation.

### 3.2 Hearing acuity and cognitive decline

No significant associations were found between continuous hearing acuity and baseline scores for verbal memory, executive function, or information processing speed (Table 2). In the longitudinal analyses, a higher score (e.g., worse hearing acuity) was associated with faster decline in all three cognitive domains. This decline was steady over time.

**TABLE 2 alz13606-tbl-0002:** Difference in baseline cognitive function and change over time per decibel (dB) of hearing loss.

	Baseline		Rate of decline from baseline to 6‐year FU		Rate of decline from baseline to 12‐year FU		Overall Hearing acuity × time^†^	
Parameter	Difference	95% CI	Difference	95% CI	Difference	95% CI	χ^2^	*P‐*Value
Verbal Memory	0.10	−0.10 to 0.31	−0.27^*^	−0.49 to −0.05	−0.96^*^	−1.27 to −0.65	37.51^*^	<0.001
Information processing speed	0.01	−0.03 to 0.05	−0.11^*^	−0.13 to −0.08	−0.24^*^	−0.29 to −0.20	128.92^*^	<0.001
Executive function	−0.05	−0.12 to 0.02	0.11^*^	0.02 to 0.20	0.35^*^	0.18 to 0.52	17.68^*^	<0.001

*Note*: Model: hearing acuity, time, hearing acuity by time, sex, age, age^2^, education level, LIBRA, LIBRA by time.

Abbreviations: CI, confidence interval; FU, follow‐up; LIBRA, Lifestyle for BRAin health index.

* *P* value < 0.05.

^†^ χ^2^, 2 degrees of freedom, of interaction between hearing acuity (continuous) and time (baseline, 6‐year, 12‐year FU).

### 3.3 Prevalent hearing loss and cognitive decline

**FIGURE 2:**
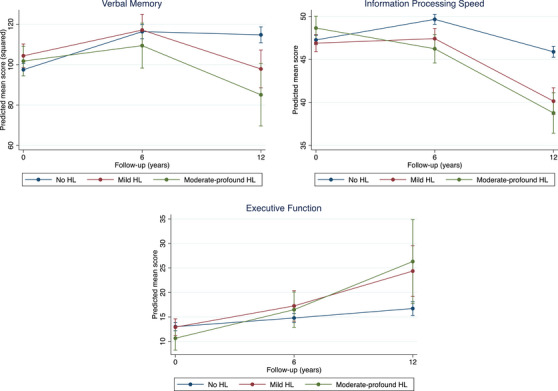
Cognitive trajectories of individuals with prevalent hearing loss and those without. Predicted mean scores are estimated marginal means of time by hearing loss (HL) status (hearing loss or no hearing loss) with all covariates fixed at their means. For the domains verbal memory and information processing speed, a higher score means a better performance. However, for executive function, a lower score means better performance

At baseline, there was no difference in baseline cognitive test scores between participants with HL (either mild or moderate to profound) and participants without HL (Table 3). Regarding rate of cognitive decline, a significant group‐by‐time interaction suggested that both participants with mild as well as participants with moderate to profound prevalent HL showed faster decline over time in verbal memory, information processing speed and executive function than the group without prevalent HL (Table 3). Comparing slopes across time showed a steady decline in all three cognitive domains (Figure 2). In addition, comparing the moderate to profound HL with the mild HL group showed that participants with moderate to profound HL had significantly higher baseline scores (indicating worse performance) on information processing speed as compared to the group with mild HL (B = 1.76, 95% confidence interval [CI] 0.16 to 3.36, *P* = .03), but did not differ from each other on verbal memory and executive function scores. The interaction with time showed no significant difference in rate of cognitive decline in verbal memory or executive function. However, results showed a significant faster rate of decline for information processing speed in the moderate to profound HL group (χ^2^ = 14.91, df = 2, *P* < .001), with a significant difference from 0 to 6 years (B = −2.94, 95% CI −4.43 to −1.44, *P* < .001), but similar rates of decline from 6 to 12 years follow‐up (B = −0.20, 95% CI −2.47 to 2.07, *P* = .86). Given that faster decline was already observed in the mild HL group and to increase power in subsquent analyses, all subsequent analyses were done for the group of combined mild to profound HL.

**TABLE 3 alz13606-tbl-0003:** Difference in baseline cognitive function and change over time in participants with prevalent hearing loss (*n* = 520) and those without (*n* = 1294).

	Baseline		Rate of decline from baseline to 6‐year FU		Rate of decline from baseline to 12‐year FU		Overall HL × time^†^	
Parameter	Difference	95% CI	Difference	95% CI	Difference	95% CI	χ^2^	*P‐*Value
**Verbal memory**								
Mild HL group	6.87	−0.10 to 13.84	−6.10	−13.15 to 0.96	−23.78^*^	−33.60 to −13.96	22.61^*^	<0.001
Moderate‐profound HL group	4.25	−4.38 to 12.87	−11.20^*^	−21.56 to −0.84	−33.95^*^	−49.83 to −18.08	17.58^*^	<0.001
**Information processing speed**								
Mild HL group	−0.37	−1.57 to 0.83	−1.87^*^	−2.79 to −0.95	−5.37^*^	−6.84 to −3.89	51.20^*^	<0.001
Moderate‐profound HL group	1.38	−0.20 to 2.97	−4.80^*^	−6.09 to −3.52	−8.50^*^	−10.85 to −6.15	66.29^*^	<0.001
**Executive function**								
Mild HL group	−0.09	−2.15 to 1.97	2.56	−1.07 to 6.19	7.74^*^	1.80 to 13.67	6.87^*^	0.032
Moderate‐profound HL group	−2.36	−5.16 to 0.45	4.05	−0.82 to 8.19	11.95^*^	3.23 to 20.68	9.27^*^	0.010

*Note*. Model: HL, time, HL by time, sex, age, age^2^, education level, LIBRA, LIBRA by time, with no prevalent HL as the reference group.

Abbreviations: CI, confidence interval; FU, follow‐up; HL, hearing loss; LIBRA, Lifestyle for BRAin health index.

* *P* value < 0.05.

^†^ χ^2^, 2 degrees of freedom, of interaction between hearing loss (yes, no) and time (baseline, 6‐year, 12‐year FU); omnibus test of the null hypothesis of no difference in rate of change over time between hearing loss groups.

### 3.4 Moderation by age group and hearing aids for prevalent hearing loss

None of the three‐way interaction models showed a modifying effect of age group (<65 years vs. ≥65 years) on the association between prevalent HL group and cognitive decline. To study the association with hearing aids, data on hearing aids at baseline was inferred from the year of possession asked to individuals still participating at 6‐year follow‐up and with available data on HL at baseline (*n* = 1369). Cognitive decline over time was compared among participants with no prevalent HL (*n* = 1021), HL without hearing aids (*n* = 38), and HL with hearing aids (*n* = 32) at baseline. No differences in cognitive decline over time were found between HL participants who had hearing aids compared to those without (Supplementary Figure 1). Relative to those without HL, participants with HL without hearing aids (χ^2^ = 21.32, df = 2; *P* < .001) and HL with hearing aids (χ^2^ = 18.19, df = 2; *P* < .001) exhibited faster decline in information processing speed. Only the former also had a significantly faster decline over time on executive function (χ^2^ = 7.88, df = 2, *P* = .02).

### 3.5 Incident hearing loss and cognitive decline

**FIGURE 3:**
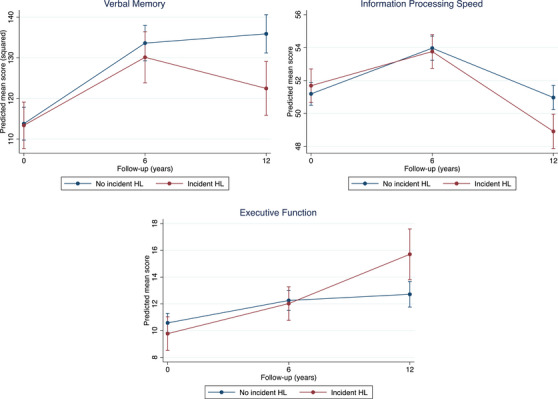
Cognitive trajectories of individuals with incident hearing loss and those without. Predicted mean scores are estimated marginal means of time by hearing loss (HL) status (hearing loss or no hearing loss) with all covariates fixed at their means. For the domains verbal memory and information processing speed, a higher score means a better performance. However, for executive function, a lower score means better performance

Table 4 and Figure 3 summarize the results for the comparisons between participants with incident HL (combined mild to profound; *n* = 360) and those without (*n* = 711). At baseline, there were no differences in cognitive test scores. Regarding rate of cognitive decline, interaction with time suggested a significant faster decline over time in all cognitive domains in the incident HL group compared to those without incident HL. Time‐stratified analyses showed that faster decline was confined to the 6‐ to 12‐year follow‐up period in all three cognitive domains (verbal memory; χ^2^ = 7.14, df = 1, *P* = .007, information processing speed; χ^2^ = 20.35, df = 1, *P* < .001, executive function; χ^2^ = 8.53, df = 1, *P* = .004) without significant decline in the initial phase (from baseline to 6‐year follow‐up).

**TABLE 4:** Difference in baseline cognitive function and change over time in participants with incident hearing loss (*n* = 360) and those without (*n* = 711)

	Baseline		Rate of decline from baseline to 6‐year FU		Rate of decline from baseline to 12‐year FU		Overall HL × time^†^	
Parameter	Difference	95% CI	Difference	95% CI	Difference	95% CI	χ^2^	*P‐*Value
Verbal memory	−0.37	−7.87 to 7.13	−3.20	−9.79 to 3.38	−13.12^*^	−20.73 to −5.51	12.03^*^	0.002
Information processing speed	0.50	−0.83 to 1.83	−0.70	−1.54 to 0.13	−2.55^*^	−3.54 to −1.56	29.16^*^	<0.001
Executive function	−0.80	−2.33 to 0.72	0.57	−1.40 to 2.54	3.80^*^	1.26 to 6.33	10.36^*^	0.006

*Note*. Model: HL, time, HL by time, sex, age, age^2^, education level, LIBRA, LIBRA by time.

Abbreviations: CI, confidence interval; FU, follow‐up; HL, hearing loss; LIBRA, Lifestyle for BRAin health index.

* *P* value < 0.05.

^†^ χ^2^, 2 degrees of freedom, of interaction between hearing loss (yes, no) and time (baseline, 6‐year, 12‐year FU); omnibus test of the null hypothesis of no difference in rate of change over time between hearing loss groups.

### 3.6 Moderation by age group for incident hearing loss

The three‐way interaction models showed that age group moderated the relationship between incident HL and information processing speed (χ^2^ = 15.26, df = 2, *P* < .001). Age‐stratified analyses showed that, similar to the main analyses, individuals with incident HL in the age group <65 showed significantly more decline over time compared to individuals without incident HL (χ^2^ = 19.73, df = 2; *P* < .001). In the age group ≥65, and contrary to expectation, individuals without incident HL showed significantly more decline over time as compared to individuals with incident HL (χ^2^ = 10.13, df = 2; *P* = .006), driven by a significant difference in the later phase, from 6 to 12‐year follow‐up (B = 6.75, 95% CI 1.54 to 11.96, *P* = .01). No moderation by age group was found for verbal memory and executive function.

### 3.7 Post hoc analyses

In post hoc analyses, we stratified the incident analyses by the time point that HL was identified (0 = never, 1 = between baseline and 6 years follow‐up, 2 = between 6 and 12 years follow‐up) to investigate whether length of HL contributes to cognitive decline. Results showed that only the 0‐6 year incident HL group, but not the 6‐12 year incident HL group, showed significantly faster decline in verbal memory and executive function compared to those without incident HL (see Supplementary material).

## 4 DISCUSSION

We investigated the association between prevalent and incident HL on 12‐year cognitive decline. Prevalent and incident HL were associated with faster cognitive decline in verbal memory, executive function, and information processing speed, independent from demographics and other modifiable risk factors. Prevalent HL showed a steady decline in cognition from baseline to 6 and 12 years, and faster decline with more severe HL, suggestive of a dose‐response association. For incident HL, decline was confined to 6‐ to 12‐year follow‐up, suggesting that onset of HL preceded or coincided with onset of decline.

Our findings support the idea that HL is a risk factor for cognitive decline and dementia. For hearing acuity, a recent study among community‐dwelling older adults showed that poorer hearing acuity was associated with a decline in verbal memory and attention over a 2‐year period, while cross‐sectionally, no associations were found.^24^ We extend this study by showing that changes in cognition become more pronounced during 12 years. Other studies found weak but significant cross‐sectional associations between hearing acuity and cognitive performance.^25^, ^26^ Differences between cross‐sectional findings might be due to sample characteristics. In our sample, mean hearing acuity was 14.3 dB, while in the studies of Bush et al.^25^ and Lin et al.,^26^ mean hearing acuity scores were 23.09 dB and 20.9 dB, respectively. Lower scores in the present study might be explained by our baseline sample being relatively younger (mean age 51.6 vs. 73.4^25^ and 64.1^26^), since hearing acuity decreases with aging. For mild and moderate to profound HL, previous longitudinal studies generally support our results that objectively measured HL is associated with accelerated cognitive decline in multiple domains.^27^, ^28^, ^29^, ^30^, ^31^ Studies examining HL with pure tone audiometry measures generally used a 25 dB threshold to define HL,^27^, ^28^, ^29^ and in the study of Deal et al.,^28^ only moderate to severe HL (>40 dB) was associated with accelerated cognitive decline. In MAAS, the association between HL and cognitive decline was already present at a 20 dB threshold. A recent study by Golub et al. supports the use of a lower threshold for defining HL by showing that individuals with a pure tone average ≤25 dB had a decreased cognitive performance on tests measuring global cognitive function, attention, information processing speed, and verbal memory.^32^

To our knowledge, incident HL has not been examined before in the context of cognitive change over time. We found that cognitive decline in incident HL was restricted to a change from 6‐ to 12‐year follow‐up, without a significant change in the first six years. Furthermore, in verbal memory and executive function, only individuals identified with HL between baseline and 6‐year follow‐up showed more decline over time compared to individuals without HL. This trajectory suggests that cognitive decline appears to evolve after onset of HL and increases gradually over time. We reported similar temporal associations between onset of dementia risk factors and cognitive decline for type 2 diabetes,^33^ hypertension,^34^ and cardiovascular disease.^35^ This possibly opens a window of opportunity for timely dementia risk management, including the use of hearing aids.

The current study showed no clear indication that having a hearing aid affects cognitive performance. The HL group with hearing aids exhibited faster decline than those without HL but similar to the HL group without hearing aids. Notably, having a hearing aid does not necessarily imply its use,^36^ and non‐use might have diluted contrasts between the two HL groups. In the UK Biobank study, the risk of dementia in people with HL who use hearing aids was similar to people without HL. However, a recent meta‐analysis showed conflicting results, and no significant association between hearing aid use and cognitive function were found in randomized controlled trials.^38^ In the recent ACHIEVE trial, no effect of hearing aids was found overall, yet a significant effect was found in people with cardiovascular risk, which might support hearing interventions for individuals at high risk of cognitive decline.^10^ Clearly, more research is warranted to substantiate this finding,^39^ but regardless of its effect on cognitive decline and dementia, treating HL has beneficial effects on quality of life, mental health, and social engagement, all by themselves considered modifiable risk factors for dementia.^11^ However, hearing aid use is very low, possibly due to stigmatization, accessibility, or lack of awareness of benefits. In MAAS, only 32 out of 520 (6.2%) participants with prevalent HL had hearing aids. Promoting the accessibility and use of hearing rehabilitation already in midlife might have important public health implications.^9^

In the age‐group <65, individuals with incident HL showed more decline over time as compared to individuals without incident HL, whereas in the opposite was found at older age. The reasons are unclear and is likely a spurious finding. In general, the study implies that the association between HL and cognitive decline is present in all age ranges, also in midlife, which is in line with the report of the Lancet Commission.^3^

Several biopsychosocial mechanisms may be implicated in the association between HL and cognitive decline.^40^ First, HL and cognitive decline might share a common underlying pathology, such as vascular changes or Alzheimer's disease affecting brain regions involved in cognition and hearing^41^ (i.e., the “common cause” hypothesis). Though we cannot exclude the risk of reversed causality (i.e., neurodegeneration causing HL), the findings that associations are apparent also in midlife, and the temporal association between onset of HL and onset of decline, make reversed causality less plausible. A second explanation is that HL affects cognition directly via impoverished sensory input leading to altered brain functional connectivity and reduced grey matter volumes,^42^ or indirectly via decreased social interaction^43^ (i.e., the “cascade” hypothesis). Another mechanism could be that individuals with HL need cognitive resources for auditory processing at the expense of other cognitive processes such as working memory^44^ (i.e., the “cognitive load” hypothesis). Although no single mechanism is likely to fully explain the relationship, the effect of rehabilitative interventions for HL, such as the use of hearing aids, is valuable for validating the proposed mechanisms. Hearing interventions should contribute to cognitive performance if the “cascade” or “cognitive load” hypotheses, which are not mutually exclusive, are valid. Contrary, the “common cause” hypothesis implies that cognitive impairment would not benefit from hearing interventions.^40^

This study has notable strengths. We used a relatively large sample with a broad age range. Hearing was assessed by pure‐tone audiometry, which is considered the gold standard, and missing data were limited to nine participants. Hearing was assessed at each follow‐up, so we were able to examine incident HL. The extensive neuropsychological test battery allowed us to investigate different cognitive domains, and the repeated assessments were sufficient to study cognitive decline over 12 years while adjusting for several relevant confounding variables in our analyses. Yet, some limitations deserve consideration. First, due to the non‐experimental nature, we cannot make causal statements about the relationship between HL and cognitive decline. Second, unmeasured or residual confounding might have impacted our results (e.g., data on apolipoprotein E [ApoE] genotype is only for a small subsample available in MAAS). Next, selection bias might have occurred due to dropout of older and more frail participants, even though maximum‐likelihood estimates from random effect models handle missing data efficiently under the missing‐at‐random assumption, based on the covariates in the model. To further minimize bias, we used inverse probability weighting, which gave more weight to observations in the analyses that had a high baseline probability of dropout. Because individuals who dropped out at 6‐year follow‐up could re‐enter at 12‐year follow‐up, it cannot be ruled out that HL was already present in some of these individuals at the timeframe between baseline and 6‐year follow‐up in our post hoc analyses. Furthermore, inferring data on hearing aids at baseline was based on self‐reported prescription dates at 6‐year follow‐up to compose a variable indicating whether individuals already possessed a hearing aid at baseline. Because of dropout of the study at 6‐years follow‐up, this could have led to exposure misclassification of baseline hearing aid possession. Also, the presence of HL might have influenced performance on cognitive tests,^45^, ^46^ although the tests used in the present study were visually administered. Last, while the sampling frame of MAAS (RNFM) and the use of sampling weights maximizes representativeness for the Dutch general population, the study has been conducted in a high‐income country and therefore results might not be generalized to countries with other socio‐demographic characteristics.

In conclusion, the present study indicates that both prevalent and incident HL predict faster cognitive decline in adults aged 24 to 82 years independent of other modifiable dementia risk factors. This association is already present in individuals with mild HL. In incident HL, cognitive decline evolves gradually over time, following the onset of HL. This possibly opens a window for risk management starting already in midlife.

## CONFLICT OF INTEREST STATEMENT

The authors declare no conflicts of interest. Author disclosures are available in the supporting information.

## CONSENT STATEMENT

All human subjects provided informed consent.

## Supporting information

Supporting Information

Supporting Information

## References

[1] Collaborators GBDDF . Estimation of the global prevalence of dementia in 2019 and forecasted prevalence in 2050: an analysis for the Global Burden of Disease Study 2019. Lancet Public Health. 2022;7(2):e105‐e125. 34998485 10.1016/S2468-2667(21)00249-8 PMC8810394

[2] Frisoni GB , Molinuevo JL , Altomare D , et al. Precision prevention of Alzheimer's and other dementias: anticipating future needs in the control of risk factors and implementation of disease‐modifying therapies. Alzheimers Dement. 2020;16(10):1457‐1468. 32815289 10.1002/alz.12132

[3] Livingston G , Sommerlad A , Orgeta V , et al. Dementia prevention, intervention, and care. Lancet. 2017;390(10113):2673‐2734. 28735855 10.1016/S0140-6736(17)31363-6

[4] Loughrey DG , Kelly ME , Kelley GA , Brennan S , Lawlor BA . Association of age‐related hearing loss with cognitive function, cognitive impairment, and dementia: a systematic review and meta‐analysis. JAMA Otolaryngol Head Neck Surg. 2018;144(2):115‐126. 29222544 10.1001/jamaoto.2017.2513 PMC5824986

[5] Yuan J , Sun Y , Sang S , Pham JH , Kong WJ . The risk of cognitive impairment associated with hearing function in older adults: a pooled analysis of data from eleven studies. Sci Rep. 2018;8(1):2137. 29391476 10.1038/s41598-018-20496-w PMC5794920

[6] Organization WH . World report on hearing. Organization WH; 2021. 10.2471/BLT.21.285643 PMC8085630 33953438

[7] Amieva H , Ouvrard C , Meillon C , Rullier L , Dartigues JF . Death, depression, disability, and dementia associated with self‐reported hearing problems: a 25‐year study. J Gerontol A Biol Sci Med Sci. 2018;73(10):1383‐1389. 29304204 10.1093/gerona/glx250

[8] Ray J , Popli G , Fell G . Association of cognition and age‐related hearing impairment in the english longitudinal study of ageing. JAMA Otolaryngol Head Neck Surg. 2018;144(10):876‐882. 30193368 10.1001/jamaoto.2018.1656 PMC6233824

[9] Maharani A , Dawes P , Nazroo J , Tampubolon G , Pendleton N , group SE‐CW . Longitudinal relationship between hearing aid use and cognitive function in older Americans. J Am Geriatr Soc. 2018;66(6):1130‐1136. 29637544 10.1111/jgs.15363

[10] Lin FR , Pike JR , Albert MS , et al. Hearing intervention versus health education control to reduce cognitive decline in older adults with hearing loss in the USA (ACHIEVE): a multicentre, randomised controlled trial. Lancet. 2023;402(10404):786‐797. 37478886 10.1016/S0140-6736(23)01406-X PMC10529382

[11] Sanders ME , Kant E , Smit AL , Stegeman I . The effect of hearing aids on cognitive function: a systematic review. PLoS One. 2021;16(12):e0261207. 34972121 10.1371/journal.pone.0261207 PMC8719768

[12] Jolles J , Houx P , Van Boxtel M , Ponds R . The Maastricht Aging Study. Determinants of cognitive aging Maastricht. Neuropsych Publishers; 1995:192.

[13] Metsemakers JF , Hoppener P , Knottnerus JA , Kocken RJ , Limonard CB . Computerized health information in The Netherlands: a registration network of family practices. Br J Gen Pract. 1992;42(356):102‐106. 1493025 PMC1371993

[14] Davis A . Hearing in adults. Whurr Publishers; 1995.

[15] Van der Elst W , van Boxtel MP , van Breukelen GJ , Jolles J . Rey's verbal learning test: normative data for 1855 healthy participants aged 24‐81 years and the influence of age, sex, education, and mode of presentation. J Int Neuropsychol Soc. 2005;11(3):290‐302. 15892905 10.1017/S1355617705050344

[16] Van der Elst W , Van Boxtel MP , Van Breukelen GJ , Jolles J . Concept shifting test. Psychol Assess. 2006;18(4):424‐432. 17154763 10.1037/1040-3590.18.4.424

[17] van der Elst W , van Boxtel MP , van Breukelen GJ , Jolles J . The Letter Digit Substitution Test: normative data for 1,858 healthy participants aged 24‐81 from the Maastricht Aging Study (MAAS): influence of age, education, and sex. J Clin Exp Neuropsychol. 2006;28(6):998‐1009. 16822738 10.1080/13803390591004428

[18] Deckers K , van Boxtel MP , Schiepers OJ , et al. Target risk factors for dementia prevention: a systematic review and Delphi consensus study on the evidence from observational studies. Int J Geriatr Psychiatry. 2015;30(3):234‐246. 25504093 10.1002/gps.4245

[19] Deckers K , Nooyens A , van Boxtel M , Verhey F , Verschuren M , Kohler S . Gender and educational differences in the association between lifestyle and cognitive decline over 10 years: the doetinchem cohort study. J Alzheimers Dis. 2019;70(s1):S31‐S41. 30507570 10.3233/JAD-180492 PMC6700651

[20] Heger IS , Deckers K , Schram MT , et al. Associations of the lifestyle for brain health index with structural brain changes and cognition: results from the maastricht study. Neurology. 2021;97(13):e1300. 34433680 10.1212/WNL.0000000000012572 PMC8480401

[21] Schiepers OJG , Kohler S , Deckers K , et al. Lifestyle for Brain Health (LIBRA): a new model for dementia prevention. Int J Geriatr Psychiatry. 2018;33(1):167‐175. 28247500 10.1002/gps.4700

[22] Coley N , Hoevenaar‐Blom MP , van Dalen JW , et al. Dementia risk scores as surrogate outcomes for lifestyle‐based multidomain prevention trials—rationale, preliminary evidence and challenges. Alzheimer's & Dementia. 2020;16(12):1674‐1685. 10.1002/alz.12169 32803862

[23] Deckers K , Köhler S , Ngandu T , et al. Quantifying dementia prevention potential in the FINGER randomized controlled trial using the LIBRA prevention index. Alzheimer's & Dementia. 2021;17(7):1205‐1212. 10.1002/alz.12281 PMC8359273 33403822

[24] Armstrong NM , An Y , Ferrucci L , Deal JA , Lin FR , Resnick SM . Temporal sequence of hearing impairment and cognition in the baltimore longitudinal study of aging. J Gerontol A Biol Sci Med Sci. 2020;75(3):574‐580. 30500877 10.1093/gerona/gly268 PMC7328201

[25] Bush ALH , Lister JJ , Lin FR , Betz J , Edwards JD . Peripheral hearing and cognition: evidence from the staying keen in later life (SKILL) study. Ear and hearing. 2015;36(4):395. 25587666 10.1097/AUD.0000000000000142 PMC4478097

[26] Lin FR . Hearing loss and cognition among older adults in the United States. J Gerontol A Biol Sci Med Sci. 2011;66(10):1131‐1136. 21768501 10.1093/gerona/glr115 PMC3172566

[27] Jiang K , Armstrong NM , Agrawal Y , et al. Associations of audiometric hearing and speech‐in‐noise performance with cognitive decline among older adults: the Baltimore Longitudinal Study of Aging (BLSA). Front Neurol. 2022;13:1029851. 36570462 10.3389/fneur.2022.1029851 PMC9784219

[28] Deal JA , Sharrett AR , Albert MS , et al. Hearing impairment and cognitive decline: a pilot study conducted within the atherosclerosis risk in communities neurocognitive study. Am J Epidemiol. 2015;181(9):680‐690. 25841870 10.1093/aje/kwu333 PMC4408947

[29] Lin FR , Yaffe K , Xia J , et al. Hearing loss and cognitive decline in older adults. JAMA Intern Med. 2013;173(4):293‐299. 23337978 10.1001/jamainternmed.2013.1868 PMC3869227

[30] Wang HF , Zhang W , Rolls ET , et al. Hearing impairment is associated with cognitive decline, brain atrophy and tau pathology. EBioMedicine. 2022;86:104336. 36356475 10.1016/j.ebiom.2022.104336 PMC9649369

[31] Stevenson JS , Clifton L , Kuzma E , Littlejohns TJ . Speech‐in‐noise hearing impairment is associated with an increased risk of incident dementia in 82,039 UK Biobank participants. Alzheimers Dement. 2022;18(3):445‐456. 34288382 10.1002/alz.12416

[32] Golub JS , Brickman AM , Ciarleglio AJ , Schupf N , Luchsinger JA . Association of subclinical hearing loss with cognitive performance. JAMA Otolaryngol Head Neck Surg. 2020;146(1):57‐67. 31725853 10.1001/jamaoto.2019.3375 PMC6865840

[33] Spauwen PJ , Kohler S , Verhey FR , Stehouwer CD , van Boxtel MP . Effects of type 2 diabetes on 12‐year cognitive change: results from the Maastricht Aging Study. Diabetes Care. 2013;36(6):1554‐1561. 23275366 10.2337/dc12-0746 PMC3661848

[34] Kohler S , Baars MA , Spauwen P , Schievink S , Verhey FR , van Boxtel MJ . Temporal evolution of cognitive changes in incident hypertension: prospective cohort study across the adult age span. Hypertension. 2014;63(2):245‐251. 24296281 10.1161/HYPERTENSIONAHA.113.02096

[35] Schievink SHJ , van Boxtel MPJ , Deckers K , van Oostenbrugge RJ , Verhey FRJ , Kohler S . Cognitive changes in prevalent and incident cardiovascular disease: a 12‐year follow‐up in the Maastricht Aging Study (MAAS). Eur Heart J. 2017. 10.1093/eurheartj/ehx365 29020327

[36] McCormack A , Fortnum H . Why do people fitted with hearing aids not wear them? Int J Audiol. 2013;52(5):360‐368. 23473329 10.3109/14992027.2013.769066 PMC3665209

[37] Jiang F , Mishra SR , Shrestha N , et al. Association between hearing aid use and all‐cause and cause‐specific dementia: an analysis of the UK Biobank cohort. Lancet Public Health. 2023;8(5):e329‐e338. 37062296 10.1016/S2468-2667(23)00048-8

[38] Yang Z , Ni J , Teng Y , et al. Effect of hearing aids on cognitive functions in middle‐aged and older adults with hearing loss: a systematic review and meta‐analysis. Front Aging Neurosci. 2022;14:1017882. 36452439 10.3389/fnagi.2022.1017882 PMC9704725

[39] Livingston G , Costafreda SG . Interventions to prevent dementia should target those at high risk. Lancet. 2023;402(10404):750‐751. 37478885 10.1016/S0140-6736(23)01472-1

[40] Uchida Y , Sugiura S , Nishita Y , Saji N , Sone M , Ueda H . Age‐related hearing loss and cognitive decline—The potential mechanisms linking the two. Auris Nasus Larynx. 2019;46(1):1‐9. 30177417 10.1016/j.anl.2018.08.010

[41] Griffiths TD , Lad M , Kumar S , et al. How can hearing loss cause dementia? Neuron. 2020;108(3):401‐412. 32871106 10.1016/j.neuron.2020.08.003 PMC7664986

[42] Fitzhugh MC , Pa J . Longitudinal changes in resting‐state functional connectivity and gray matter volume are associated with conversion to hearing impairment in older adults. J Alzheimers Dis. 2022;86(2):905‐918. 35147536 10.3233/JAD-215288 PMC10796152

[43] Evans IEM , Martyr A , Collins R , Brayne C , Clare L . Social isolation and cognitive function in later life: a systematic review and meta‐analysis. J Alzheimers Dis. 2019;70(s1):S119‐S144. 30372678 10.3233/JAD-180501 PMC6700717

[44] Tun PA , McCoy S , Wingfield A . Aging, hearing acuity, and the attentional costs of effortful listening. Psychol Aging. 2009;24(3):761‐766. 19739934 10.1037/a0014802 PMC2773464

[45] Nichols E , Deal JA , Swenor BK , et al. Assessing bias in cognitive testing for older adults with sensory impairment: an analysis of differential item functioning in the Baltimore longitudinal study on aging (BLSA) and the atherosclerosis risk in communities neurocognitive study (ARIC‐NCS). J Int Neuropsychol Soc. 2022;28(2):154‐165. 33896441 10.1017/S1355617721000400 PMC8546003

[46] Dupuis K , Pichora‐Fuller MK , Chasteen AL , Marchuk V , Singh G , Smith SL . Effects of hearing and vision impairments on the montreal cognitive assessment. Neuropsychol Dev Cogn B Aging Neuropsychol Cogn. 2015;22(4):413‐437. 25325767 10.1080/13825585.2014.968084

