# Supplementary material for: Cognitive change in prevalent and incident hearing loss: The Maastricht Aging Study
Source: Alzheimers Dement. 2024 Jan 18;20(3):2102–12. doi: 10.1002/alz.13606 (PMC10984489; doi:10.1002/alz.13606)
Supplement: Supplementary file 1 — Supporting Information [file ALZ-20-2102-s002.docx]

**Supplementary Table 1. Operationalization of the LIBRA index in MAAS.**

| **Factor** | **Operationalization** | **Weight** |
| --- | --- | --- |
| Coronary heart disease | Presence of cardiac rhythm disorders, chest pain/angina pectoris, heart attack, heart insufficiency, bypass surgery, and/or self-reported presence of heart disease. | +1.0 |
| Diabetes (type-2) | Use of diabetes medication, diabetes diagnosis, and/or self-reported presence of diabetes. | +1.3 |
| Hypercholesterolemia | Use of cholesterol medication, and/or self-reported presence of high cholesterol. | +1.4 |
| Hypertension | Average (of 5 assessments at the research centre) systolic blood pressure ≥ 140 mmHg, and/or average (of 5 assessments at the research centre) diastolic blood pressure ≥ 90 mmHg [1], and/or current antihypertensive medication use. In case of missings, self-reported presence of hypertension was used. | +1.6 |
| Depression | The depression part of the Symptom Check List (SCL 90) was used [2], divided into quartiles. Participants in the highest quartile were assigned in the risk group. | +2.1 |
| Obesity | Body mass index (BMI) ≥ 30 kg/m2 calculated from physical examination at the research centre or (in case of missing BMI) a waist circumference of >88 cm for women and >102 cm for men. [3] | +1.6 |
| Smoking | Self-reported current smokers or non-smokers. | +1.5 |
| Low-to-moderate alcohol use | Self-reported alcohol intake. Low to moderate alcohol use was defined as ≤7 alcoholic consumptions per week. | -1.0 |
| Physical inactivity | Self-reported hours per day up and about, divided into tertiles. Participants in the lowest tertile were assigned as physically inactive. In case of missings, self-reported hours per week spent on exercising was used. Participants who did not adhere to the ≥150 minutes per week of exercising were assigned as physically inactive. | +1.1 |
| High cognitive activity | Sum of self-reported average hours per week spent on reading (books, magazines, newspapers) and mind games (chess, checkers, puzzles), divided into tertiles (low/medium/high). Participants in the highest tertile were categorized as cognitively active. | -3.2 |
| Renal dysfunction | Presence of kidney disease (kidney stones excluded). | +1.1 |
| Total theoretical LIBRA range |  | -4.2 to +12.7 |

NOTE. Abbreviations: LIBRA, LIfestyle for BRAin health; MAAS, Maastricht Ageing Study; WHO, World Health Organization.

References

[1] Guidelines Subcommittee (1999) 1999 World Health Organization-International Society of Hypertension Guidelines for the Management of Hypertension. J Hypertens 17, 151-183.

[2] Arrindell WA, Ettema JHM. 1986. SCL-90: A Multidimensional Indicator of Psychopathology. Swets & Zeitlinger: Lisse, The Netherlands.

[3] World Health Organization (2008) Waist circumference and waist–hip ratio: Report of a WHO expert consultation. World Health Organization.

**Supplementary Figure 1. Cognitive trajectories of individuals without hearing loss (n=1021), hearing loss without hearing aids (n=38) and hearing loss with hearing aids (n=32).
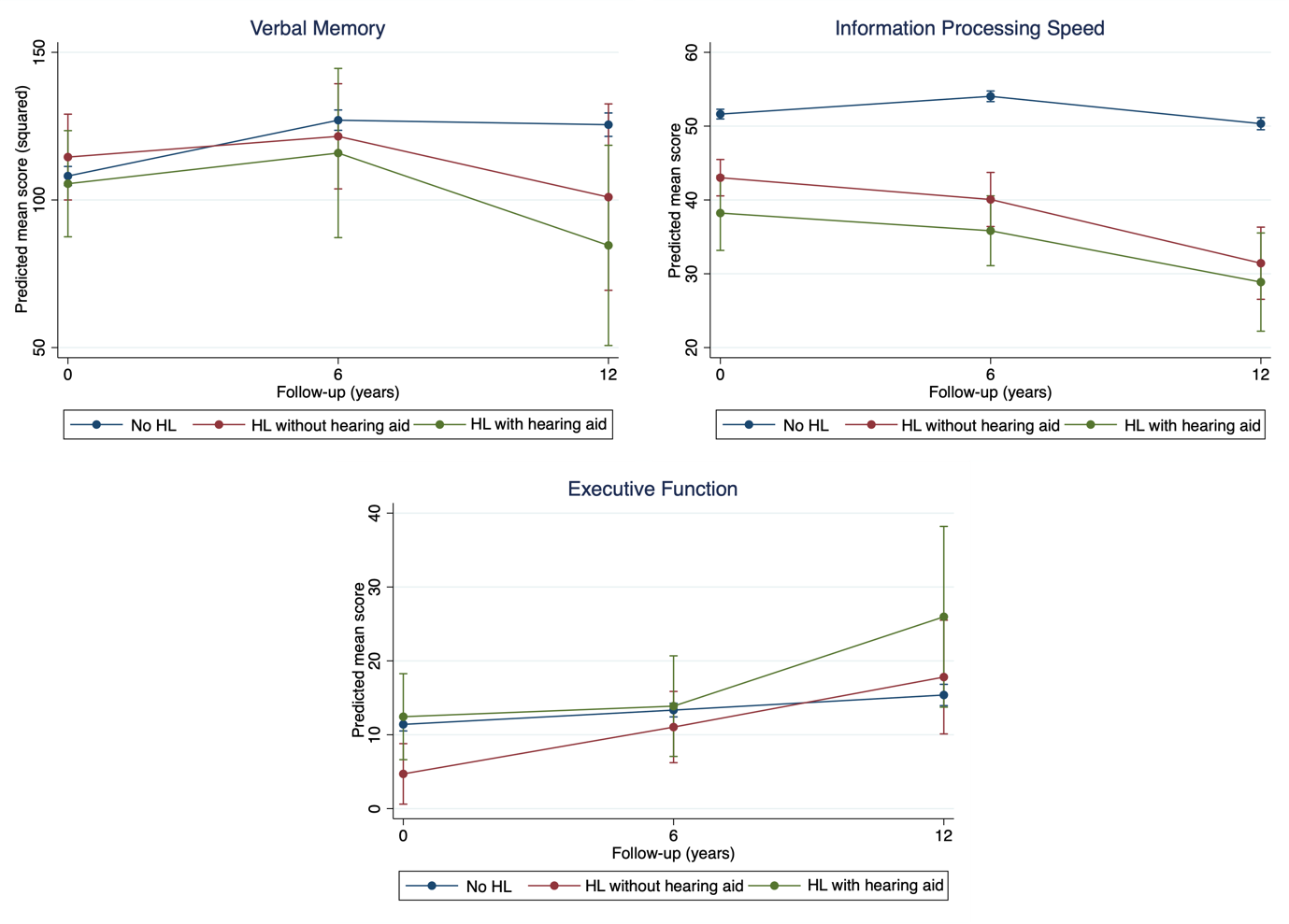
**

NOTE. Predicted mean scores are estimated marginal means of time by hearing aid status with all covariates fixed at their means. For the domains verbal memory and information processing speed, a higher score means a better performance. However, for executive function, a lower score means better performance.

**Supplementary material: post hoc analyses**

Post hoc analyses of participants without incident HL (n=711), incident HL identified between 0-6 years follow up (n=202) and incident HL identified between 6 and 12 years follow up (n=158).

- Relative to those without incident HL, participants with incident HL identified between 0-6 years follow up exhibited faster decline in memory (χ^2^=15.53, df=2, p<.001), information processing speed (χ^2^=20.19, df=2, p<.001) and executive function (χ^2^=9.31, df=2, p=.009)
- Participants with incident HL identified between 6-12 years follow up only exhibited faster decline in information processing speed (χ^2^=16.21, df=2, p<.001) as compared to individuals without incident HL, but not in memory (χ^2^==1.68, df=2, p=.4321) or executive function χ^2^=2.16, df=2, p=.339)
- Comparing the 0-6 incident HL group with the 6-12 incident HL group showed no significant difference in verbal memory (χ^2^=5.30, df=2, P=.07), information processing speed (χ^2^=1.04, df=2, p=.595) or executive function (χ^2^=3.34, df=2, p=.188)

NOTE. Predicted mean scores are estimated marginal means of time by incident HL group with all covariates fixed at their means. For the domains verbal memory and information processing speed, a higher score means a better performance. However, for executive function, a lower score means better performance.
